# “Living a normal life”: a qualitative study of patients’ views of medication withdrawal in rheumatoid arthritis

**DOI:** 10.1186/s41927-019-0070-y

**Published:** 2019-06-13

**Authors:** Kenneth F. Baker, John D. Isaacs, Ben Thompson

**Affiliations:** 1 Musculoskeletal Research Group, Institute of Cellular Medicine, Newcastle University, Framlington Place, Newcastle upon Tyne, NE2 4HH UK; 2 Musculoskeletal Unit, Newcastle upon Tyne Hospitals NHS Foundation Trust, Newcastle upon Tyne, UK

**Keywords:** Rheumatoid arthritis, DMARD, Remission, Qualitative, Withdraw, Cessation

## Abstract

**Background:**

Withdrawal of disease-modifying anti-rheumatic drugs (DMARDs) once disease remission is achieved is endorsed by current international rheumatoid arthritis (RA) management guidelines. However, very little data exists concerning patients’ views of this practice. In this qualitative study, we aimed to explore patients’ perspectives on DMARD withdrawal in the setting of established RA.

**Methods:**

In this qualitative interview study, patients with stable established RA were recruited from rheumatology outpatient clinics at a large UK teaching hospital. The perceived advantages and disadvantages of DMARDs and views on DMARD withdrawal were explored in semi-structured interviews. Interview transcripts were analysed using standard qualitative techniques to construct an analytical framework.

**Results:**

Thirteen participants (8 female, median [IQR] age 65 [61–73]) expressed their views of DMARD treatment in the context of their “normal lives”. For some patients, disadvantages such as medication side-effects and the inconvenience of safety monitoring were sufficient hindrances to their lifestyle to justify DMARD withdrawal. However, patients who were vulnerable to loss of physical function, or who had prior experience of severe rheumatoid arthritis, expressed a strong preference against DMARD withdrawal, viewing the potential for increased pain and future disability as unacceptable risks.

**Conclusions:**

Patients view DMARD withdrawal in the context of either restoring or threatening their “normal lives”. In this model, social and personal factors play a crucial role in influencing patients’ opinions of DMARD therapy beyond a simple consideration of medication side-effects alone. A formulaic approach to DMARD withdrawal determined and imposed by clinicians would not be successful. Instead, the discussion of DMARD withdrawal should take place with the identification of patients’ priorities and in the context of their personal disease experiences.

**Trial registration:**

clinicaltrials.gov (NCT02064400), retrospectively registered 17 February 2014.

**Electronic supplementary material:**

The online version of this article (10.1186/s41927-019-0070-y) contains supplementary material, which is available to authorized users.

## Background

The traditional view of rheumatoid arthritis (RA) as a chronic destructive arthritis where pain, stiffness and disability are inevitable for all patients is now obsolete. Remission is now a realistic target of treatment for many patients through the use of modern disease modifying anti-rheumatic drugs (DMARDs), particularly when used in combination and early in the course of the disease [1]. Nevertheless, the use of DMARDs is associated with significant burdens including the risk of toxicity and the inconvenience posed by regular dosing and monitoring schedules [2]. With significant numbers of patients now achieving remission, patients and their clinicians are increasingly faced with a therapeutic conundrum: when is it appropriate to withdraw DMARD therapy once remission is achieved?

It is important to recognise the dilemma facing patients when confronted with the prospect of DMARD withdrawal. Several studies have explored patients’ beliefs and experiences surrounding DMARD therapy in RA and the crucial effects of these upon medication adherence [3–6]. Much has also been published on the topic of normalisation in long-term disease – originally described by Strauss and Glaser [7], and more recently defined by Morse et al. [8] as the “strategies designed to minimise the impact of (or accommodate) an illness or related disability or both”. However, there is a paucity of studies that have addressed the importance of patients’ opinions and concerns regarding the decision to withdraw DMARD therapy once remission has been achieved.

Longitudinal cohort data in early RA suggest that up to 15% of all patients can achieve DMARD-free remission [9], with prospective clinical trials demonstrating maintenance of remission in up to half of patients after complete withdrawal of DMARD therapy [10, 11], albeit at the risk of disease flare. [12] Guidelines from the European League Against Rheumatism [13] and American College of Rheumatology [14] support the concept of DMARD withdrawal for patients with RA who achieve remission. Nevertheless, considerable uncertainty exists, not least due to the absence of robust biomarkers of remission and a lack of consensus regarding the optimal strategy for DMARD withdrawal [15, 16].

In this study we present an analysis of the issues surrounding DMARD withdrawal from the patient perspective. Through the use of semi-structured interviews we aimed to identify the perceived advantages and disadvantages of DMARD therapy, and the extent to which these and other factors influence patients’ opinions of DMARD withdrawal.

## Methods

### Study design

We conducted a qualitative study of patients’ views on DMARD therapy and DMARD withdrawal using semi-structured interviews. The study was designed to assess the feasibility of a future clinical trial of DMARD withdrawal, and to inform the consent and counselling process required.

### Participants

Patients were consecutively recruited to the study from routine outpatient clinic attendances face-to-face by their rheumatology clinician in a single UK hospital. Eligible patients were identified as those who had a clinical diagnosis of RA controlled by DMARDs, with symptom onset at least 12 months previously and stable disease defined as no change in DMARD therapy or use of corticosteroids in the previous 6 months before recruitment. No patients receiving biologic therapy had been recruited after the first 10 interviews, and thus protocol-specified purposive sampling [17] was used to specifically recruit additional patients receiving biologic agents. Study recruitment was stopped when saturation [18] of themes was reached after 13 patients (i.e. when no new themes emerged from consecutive interview analyses), reflecting the anticipated target of 10–15 interviews as specified in the study protocol.

All patients were assessed for joint pain and swelling on the day of interview by the same clinician (KB). This was combined with their most recent erythrocyte sedimentation rate (ESR) measurement to calculate their Disease Activity Score in 28 Joints (DAS28) [19]. A retrospective clinical notes review was conducted to assess cumulative fulfilment of the 2010 American College of Rheumatology/European League Against Rheumatism (ACR/EULAR) diagnostic criteria for RA [20], with all but one patient (Patient M) satisfying these criteria. Nevertheless, this patient had a clinical diagnosis of established RA confirmed by a consultant rheumatologist and had received long-term DMARD therapy, and thus satisfied our study inclusion criteria.

### Data collection

Semi-structured patient interviews (‘Patient interview schedule’, online Additional file 1) were developed to address two broad areas, namely: 1) the benefits, negative aspects and concerns surrounding DMARD therapy, and 2) views of patients concerning complete DMARD withdrawal within a clinical research setting (the latter of which to inform future clinical trial design). Interviews were conducted by the same researcher (KB) in a private rheumatology outpatient consulting room. Following a consent process and explanation of the interview format, patients were asked to discuss advantages and disadvantages of DMARD therapy and opinions regarding theoretical DMARD withdrawal in the setting of the aforementioned future clinical trial. Interview schedule questions were used as initial prompts for discussion, with patients encouraged to raise and discuss more widely the issues relevant to their own personal circumstances and experiences.

### Data analysis

Audiotape recordings of a median (range) duration of 15.2 (7.9 to 27) minutes were transcribed verbatim using conversation notation [21]. In a qualitative content analysis approach, [22] transcripts were analysed and coded line-by-line by KB and BT, and coding practices reviewed and agreed. Interview themes were explored by KB/BT using standard qualitative techniques including constant comparison, deviant case analysis and memoing to create an analytical framework (Additional file 2: Figure S1 and Additional file 3: Figure S2, online Additional files).

### Rigor and accuracy of the qualitative study

KB is a male rheumatology clinical researcher with experience in the diagnosis and management of RA, who designed and conducted the study as part of a supervised Masters degree. BT is a male rheumatology consultant with extensive experience of qualitative research including his own qualitative MD research degree, and supervision of qualitative research studies at Masters, Doctoral and post-Doctoral levels. Interview transcripts were checked against the original audio recordings and their accuracy verified by KB. Once the analytic framework was developed, this was reviewed with reference to the transcripts and agreed between all researchers. This study adheres to the COREQ (COnsolidated criteria for REporting Qualitative research) standards [23].

### Ethical considerations

The study was approved by a research ethics committee (National Research Ethics Service East of England – Cambridge Central Research Ethics Committee [13/EE/0459]). All patients provided written informed consent before participation, which included permission to reproduce anonymised verbatim quotations from interview transcripts. Research was conducted in accordance with the Declaration of Helsinki [24].

## Results

### Characteristics of patient cohort

Thirteen patients were interviewed over a three-month period, the majority with established RA of several years duration (Table 1). Methotrexate monotherapy was the most common DMARD regimen; 10/13 patients achieved a DAS28 < 2.6 in keeping with disease remission.

**Table 1:** Patient demographics and clinical details

Demographic / clinical data	Value
Number of patients recruited	13
Female [%]	8 [53%]
Age: median [IQR, range] years	65 [61–73, 44–85]
Time since RA diagnosis: median [IQR] years	10 [3–13]
RhF and/or ACPA positive: n [%]	9 [69%]
Current use of methotrexate: n [%]	11 [85%]
Current use of hydroxychloroquine: n [%]	1 [8%]
Current use of sulfasalazine: n [%]	2 [15%]
Current use of biologics: n [%]	2 [15%]
DAS28: median [IQR]	1.56 [1.35–2.44]

ACPA: anti-citrullinated peptide antibody: IQR: interquartile range, RhF: rheumatoid factor

### Advantages of DMARD therapy

Participants identified a wide range of advantages of receiving DMARD therapy (Table 2). In seven cases, these centred on the alleviation of physical symptoms such as pain, swelling and stiffness. For these patients, this was seen as helping to maintain their everyday physical function.

*It [methotrexate and sulfasalazine] keeps you mobile, you know? Your joints are flexible, you know, you’re able to do things that you perhaps wouldn’t be able to, you know? Just simple everyday things.* (Patient A, 70-74 yrs., RA for 11 years).

**Table 2:** The advantages and disadvantages of disease modifying anti-rheumatic drug (DMARD) therapy as perceived by patients

Advantages of DMARDs	Disadvantages of DMARDs
Alleviation of physical symptoms of RA (i.e. pain, swelling and stiffness)	Side-effects of medication
Maintain mobility: 1. For everyday physical functions 2. For employment	Practical issues with taking medication: 1. Extra appointments for blood monitoring tests 2. Ordering and taking tablets 3. Difficulties in transporting medication abroad
Prevent future deterioration and deformity	Complication of healthcare provided by non-rheumatology specialists

The ability to keep performing daily physical acts was often expressed in terms of “maintaining mobility”, “keeping steady” and ultimately “living a normal life”. In addition to permitting a ‘normal life’ through maintenance of daily activities, four patients identified DMARDs as restoring a holistic state of normality.

*KB: What do you feel as the benefit to you of etanercept?*

*Pt D: Living a normal life. Mobility, everything really that I couldn’t do when I had flare ups, which I don’t have now. It enables me to walk without pain. To do all activities without pain. A different way of life.* (Patient D, 65-69 yrs., RA for 14 years).

All patients could identify symptomatic and functional benefits of DMARD therapy. However, five patients appeared to have little expectation of DMARDs improving their own current condition. Instead, DMARD therapy was highly valued by these patients in terms of preventing future deterioration of their symptoms.

*I don’t particularly feel there has been any correction or improvement [with taking DMARDs]. But certainly I feel no worse. So like a status quo.* (Patient F, 60-64 yrs., RA for 3 years).

The fear of future symptom burden and physical disability was a strong recurring theme throughout the interview process. In some cases this concern also extended to the possibility of future physical deformities, recognised as both unsightly and limiting physical ability, and was a powerful motivation to continue DMARD therapy.

*I don’t wanna believe that, they [my joints] would be deformed or owt like that, you know what I mean? So, you know, if you can keep everything steady that’s great for me. The last thing that I want is to be like I have when I’ve like seized up in my fingers, and stuff like that. So, if methotrexate can sort of prevent that, I’m happy to take it.* (Patient E, 40-44 yrs., RA for 1 year).

In summary, although a wide range of advantages of DMARDs were identified by patients in our cohort, these were framed within the context of their ability to lead a “normal life”. Many patients were fearful of future symptom burden and disability, which was sufficient motivation to continue therapy even in the absence of any short-term symptomatic relief or functional improvement.

### Disadvantages of DMARD therapy

The physical side-effects of medications were a strongly cited disadvantage of DMARD therapy (Table 3), though individual patients described different mechanisms to deal with this potential threat. For some patients, the balance of side-effects versus effective control of RA appeared to be an important factor in the decision to commence DMARD therapy. Thus for these patients, their DMARD therapy represented a favourable balance of benefit versus risk. However, five patients chose to overlook the potential side-effects when taking DMARDs and instead delegated this concern to another individual such as their doctor or a family member.

**Table 3:** Summary of the side effects of disease modifying anti-rheumatic drug (DMARD) therapy identified by patients within the study

Organ system	Side-effect
Gastrointestinal	Nausea, vomiting, heartburn, hepatitis, bowel disturbance
Skin	Rash, bruising, stinging at injection site (biologics), hair loss
General health	Fatigue, mood swings, weight gain
Miscellaneous	Increased infection risk, blood cell abnormalities, change in urine colour, possible increased cancer risk

*Pt I: Me* [colloq. *My*] son *who has the same, said “Have you read the leaflet? It causes* terrible *problems!” and I said “Well I haven’t read the leaflet and it hasn’t caused me any problems”. But he is like me husband, he reads every little last detail on the leaflet.*

*KB: Yes, yes. Did you get a leaflet at the time?*

*Pt I: I did, yeah.*

*KB: Yes, yes. And why didn’t you want to read through all of it? Was it because …*

*Pt I: Because I never do. I never read leaflets.*

*KB: Is that because it, because you find it …*

*Pt I: I think it would put you off.* (Patient I, 75-79 yrs., RA for 13 years).

The need to attend extra appointments for blood monitoring tests, order tablets from pharmacy and difficulties in transporting medication abroad were pragmatic disadvantages highlighted by six patients as interfering with a “normal” lifestyle.

*If I go on holiday, I’ve got to make sure that I have a fridge where I go. I mean I’ve just been away, and it [adalimumab dose] was in the middle of our holiday, which was badly planned really. It’s just thinking of ways to keep this injection at the right temperature.* (Patient J, 65-69 yrs., RA for 11 years).

*Having to order prescriptions every month, you know, go and collect them, put your tablets out every evening for the next day* (Patient A, 70–74 yrs., RA for 11 years).

In addition, one patient who received biologic therapy also reported that DMARD therapy caused complications with their healthcare, particularly the management of comorbidities by non-rheumatology specialists who were unfamiliar with their DMARD therapy.

*They’re not aware, you know, I don’t think that anybody outside this field is aware of the type of medication you take, really.* (Patient J, 65-69 yrs., RA for 11 years).

In summary, patients could identify disadvantages to DMARDs in terms of barriers to their “normal” lifestyle and physical side-effects. For some patients (exemplified by the quotations from patients F & E above), the risk of continued symptoms, physical limitation and joint deformities were viewed as sufficient to justify DMARD therapy despite the potential disadvantages. In contrast, others (such as patient I above) chose to overlook the potential risks and focus exclusively upon the benefits of treatment as a means to justify DMARD therapy.

### DMARD withdrawal

Patients could relate to the dilemma of DMARD withdrawal in the setting of remission and the uncertainty that this poses.

*‘Cause you wonder if you keep on taking stuff like, whether, it’s, you’re just taking it, and would you be alright without it, you know? You, you don’t know, because you just keep taking it all the time.* (Patient J, 65-69 yrs., RA for 11 years).

Patients generally expressed a desire to reduce their medication, driven by concerns regarding the potential toxicity of long-term DMARD therapy. Nevertheless, these concerns tended not to focus on specific side-effects but instead as an ill-defined notion of DMARDs as deleterious to health. Two patients believed this effect to be cumulative and specific to long-term DMARD use, and hence recognised DMARD withdrawal as advantageous in limiting this effect.

*I don’t know what the long term, if any long-term side effects happen with this I’ve no idea, you know, not that I remember anyway. It’s always better to stop something if you don’t need it.* (Patient H, 55-59 yrs., RA for 1 year).

*KB: What would be the benefits for you do you think of stopping methotrexate?*

*Pt K: I think, just less toxicity for your body really isn’t it?* (Patient K, 50-54 yrs., RA for 10 years).

In addition, patients could also identify further benefits of DMARD withdrawal in terms of restoring a degree of normality to their lifestyle. A strong recurring theme throughout the interviews was a desire to reduce the burden of prescribed medication, which was viewed as a substantial benefit in its own right.

*Pt C: I would love to come off them. I would love to come off all the tablets that I’m on.*

*KB: And, and what would your reasons be for wanting to come off of methotrexate?*

*Pt C: Just because I’m sick of taking tablets.* (Patient C, 85-89 yrs., RA for 2 years).

For four patients, the DMARD dosing and monitoring schedules posed a substantial barrier to their lifestyle. For these patients, DMARD withdrawal offered a means by which they could achieve a freedom from the constraints of DMARD monitoring and hence live a more “normal life”.

*Every now and then I go away for a reasonably longer period because I’m retired now. So I can go away for two or three months for example. So, I’m stopped from doing that because of having to consider this blood monitoring situation. And then, I cannot go away more than 6, 7 weeks. Okay? And it would have to coincide with this gap, this window, which is due to blood monitoring tests. If … I was able to do without the medication for a longer period, then that may give you back that freedom.* (Patient F, 60-64 yrs., RA for 3 years).

The majority (9/13) of patients who were interviewed would consider withdrawal of DMARD therapy, although four patients would never consider stopping DMARD treatment. Particularly important amongst this group was their prior experience of RA. Personal experience of previously difficult-to-control or severe RA was a strong disincentive to discontinue DMARDs. Furthermore, experience of friends or family with severe and deforming RA was also a strong factor against DMARD cessation. For one patient (Patient M), the act of taking DMARDs was seen as a positive control mechanism that could be used to prevent future deterioration of their RA. For this patient, the prospect of DMARD withdrawal represented a loss of control over their disease with consequent worry of future irreversible joint damage, reinforced by the severe RA they had witnessed within their own family.

*Pt M: I think it’s reassuring when you’re actually doing something, when you’ve got a history of it [RA] in the family and you know how worse it can get. I don’t want to get to that point where, I’m just, you know allowing it to progress.*

*KB: Right. So it’s actually sort of, taking the tablet almost feels like you are doing something to help control things?*

*Pt M: Yes. So to come off of it would be worrying.* (Patient M, 60-64 yrs., RA for 5 years).

In addition, patients also considered the consequences of a deterioration in RA control in terms of their individual social circumstances when contemplating DMARD withdrawal, with those in current employment or with dependent others attributing greater importance to this.

*My main concern [about stopping DMARDs] is obviously if my symptoms are sort of untreated though, and continued. I don’t want to get deformities, other than what I’ve got at the minute. It could affect my job dramatically, bearing in mind we’ve all got to work longer. You know, it’s pretty hard to hold a saw or a hammer if your fingers are pointing in all directions.* (Patient E, 40-44 yrs., RA for 1 year).

In summary, patients expressed a strong desire to reduce their DMARD medication, driven by the hindrance it places upon their “normal” lifestyle and concerns regarding its potential toxicity. Nevertheless, the threat of loss of control of RA activity upon DMARD withdrawal was also considered and was prioritised by those with previous experience of severe RA or vulnerability to loss of physical function. A summary of the issues identified in the patient interviews surrounding DMARD cessation is presented in Table 4.

**Table 4:** Overview of the themes arising from patient interviews surrounding potential withdrawal of disease-modifying anti-rheumatic drug (DMARD) therapy

Theme	Comments
Uncertainty after DMARD withdrawal	Uncertainty and unpredictability of developing flare of RA after DMARD withdrawal
Getting rid of unnecessary medication	Benefits of stopping “unnecessary medication” in terms of avoidance of toxicity and the need for blood monitoring
Feeling of loss of control over disease	DMARDs as a “weapon” that fights disease, without which patients feel a loss of control over their RA
Previous disease experiences	Personal or family/friend experience of treatment-resistant or deforming RA is a strong disincentive to DMARD withdrawal
Social circumstances	Patient less likely to consider DMARD withdrawal if their social circumstances make them vulnerable to disease flare and periods of reduced physical function – e.g. caring for dependent family member, manual worker.

## Discussion

The patients within this study expressed views of DMARD therapy in terms of either enablement or hindrance of their daily routine and ability to live a “normal life”, a strong theme in our analysis. One strategy of normalisation is to downgrade expectations of a “normal” state, illustrated by the relatively modest definitions of normality described by many patients in this study – to walk and wash oneself without pain, for example. Nevertheless, medication represents an important tool to reduce the burden of living with RA; indeed, minimisation of the personal impact of RA and a return to normality have been demonstrated to be central priorities in recent studies of patient-reported expectations of DMARD therapy [25] and RA remission [26].

These patients with established RA identified advantages of DMARD therapy in terms of reduced symptoms, maintenance of mobility or prevention of future deterioration. Nevertheless, the same patients also recognised disadvantages to DMARD therapy including medication side-effects, blood safety monitoring and the practicalities of taking regular medication. This range of views regarding DMARD therapy is comparable to previous studies of patient views of long-term DMARD therapy in RA [4, 5].

A notable recurring theme in our study was a strong desire of many patients to reduce the amount of medication they received. Indeed, a preference towards drug minimisation has been described previously in studies of long-term illness both within and outside the setting of RA [4]. For many patients in this study, a preference towards DMARD withdrawal was underpinned by concern regarding the potential long-term toxicity of DMARDs. Nevertheless, several patients in our study overlooked these potential side-effects yet still favoured DMARD withdrawal, supported by a wider consideration of the impact of DMARD therapy upon their individual lives. Indeed, in a recent study of patients’ views of DMARD escalation in RA, additional personal and sociodemographic factors such as current employment and caring roles were found to play a crucial role in the decision-making process beyond a simple consideration of medication side-effect profile [27].

In a previous qualitative study of 20 patients with RA, Markusse et al. [28] observed a range of both positive and negative emotions of patients when considering the prospect of DMARD withdrawal. Many of these emotions paralleled those expressed by patients in our study and included happiness and relief at avoiding regular medication tempered by fear of the return of RA symptoms and arthritis progression. An analysis of the impact of previous disease experiences and social circumstances was not included, although our study also suggests that these are important additional factors that can crucially influence patients’ views of DMARD withdrawal.

Based upon the results of our study, we propose a framework whereby patients consider the impact of DMARD withdrawal in the context of either facilitating or hindering their “normal life”. For many patients, the disadvantages of DMARD therapy pose significant lifestyle hindrances and support a preference towards DMARD withdrawal. Nevertheless, for some patients the risk of deterioration in their RA due to medication withdrawal is sufficient motivation to continue DMARD therapy. Crucially, patients who are vulnerable to loss of physical function – for example, by virtue of their employment or dependent others – or who have previous experience of severe RA, prioritise this risk and thus favoured DMARD continuation. Furthermore, it is important to note that all of the patients in this study expressed a clear preference either for or against DMARD withdrawal. This highlights the importance of an individualised approach when addressing the issue of DMARD minimisation, and suggests that simple decision pathways driven by assessments of disease activity alone would not be successful.

There are several limitations to this study, not least that participants were asked to discuss their views of a theoretical withdrawal of DMARD therapy in a research environment, which may differ from their actions in practice. The interview duration was relatively short, and it is possible that longer discussions may have identified additional more complex themes. Furthermore, patients in this study were specifically asked to discuss stopping DMARD therapy in relation to a proposed future trial of complete DMARD withdrawal, and may have responded differently if asked to consider partial tapering of therapy. Nevertheless, we believe the issues raised are of relevance to clinicians and patients in the setting of both withdrawal and tapering of treatment. The limited ethnic diversity of patients attending our rheumatology department, combined with the relatively advanced age of the study cohort, may affect the generalisability of the results to other populations. Furthermore, interviewing by a clinician in a hospital setting may have influenced the range of opinions expressed by participants who may be less willing to express opinions contrary to those of their rheumatology team. [23] This study was a pilot study to inform the design of an imminent future clinical trial of DMARD withdrawal – a further additional pilot of this study was not performed, though it is possible that this may have helped to further refine the methodology and analyses presented herein.

## Conclusions

With the significant rates of remission in RA observed with modern DMARD therapy, the question of when and how to reduce the burden of immunosuppressive medication in this group of patients is of increasing importance. The withdrawal of DMARDs represents the later stages of what can often be a long and challenging illness journey, with patients’ opinions heavily influenced not only by their current personal circumstances but also by their previous experiences. Our study offers this unique insight in order to help clinicians address patients’ concerns in this fundamentally individualised and shared decision-making process.

## Additional files

Additional file 1: Patient interview schedule. Semi-structured patient interview schedule. (PDF 308 kb)

Additional file 2: **Figure S1.** Hierarchial tree of analytic themes relating to advantages and disavantages of DMARD therapy. (PDF 129 kb)

Additional file 3: **Figure S2.** Hierarchical tree of analytic themes relating to withdrawal of DMARD therapy. (PDF 129 kb)

## Data Availability

The authors declare that the data supporting the findings of this study are available within the article and its supplementary information. This article has been written with reference to COREQ (COnsolidated criteria for REporting Qualitative research) standards.

## Abbreviations

ACR: American College of Rheumatology
DAS28: Disease activity score in 28 joints
DMARD: Disease-modifying anti-rheumatic drug
ESR: Erythrocyte sedimentation rate
EULAR: European League Against Rheumatism
RA: Rheumatoid arthritis
