# Supplementary material for: “Living a normal life”: a qualitative study of patients’ views of medication withdrawal in rheumatoid arthritis
Source: BMC Rheumatol. 2019 Jun 13;3:2. doi: 10.1186/s41927-019-0070-y (PMC6567658; doi:10.1186/s41927-019-0070-y)
Supplement: Supplementary file 1 — Patient interview schedule. Semi-structured patient interview schedule. (PDF 308 kb) [file 41927_2019_70_MOESM1_ESM.pdf]

Semi-structured patient interview schedule

The following semi-structured interview schedule is reproduced from the study protocol:

Introduction

Define with patient their current DMARD therapy and that these questions relate solely to their DMARD treatment and not any other medications they may be taking. DMARD therapy is defined as any non-biological or biological disease modifying anti-rheumatic drug currently being prescribed to the patient. Interviews should be initially directed by the questions listed below, with further questioning to explore any issues identified permissible.

Start audiotape

1. What are the benefits for you in continuing to take your DMARD therapy?
2. What are the negative aspects of taking regular DMARD therapy for you?
3. Do you have any concerns about side-effects of DMARD therapy? If yes - are there any specific side-effects you have read about that concern you?

We plan to start a clinical trial that aims to identify blood tests that may predict patients with rheumatoid arthritis who potentially can stop their DMARD treatment with a low risk of their arthritis flaring. This study would involve patients with stable arthritis, who would completely stop their DMARD treatment and be monitored for 6-12 months thereafter.

1. Would you have any concerns about stopping your DMARD treatment? If so, what would they be?

Stop audiotape
