# Supplementary material for: “Living a normal life”: a qualitative study of patients’ views of medication withdrawal in rheumatoid arthritis
Source: BMC Rheumatol. 2019 Jun 13;3:2. doi: 10.1186/s41927-019-0070-y (PMC6567658; doi:10.1186/s41927-019-0070-y)

Supplementary Figure S1 – Hierarchial tree of analytic themes relating to advantages and disavantages of DMARD therapy.

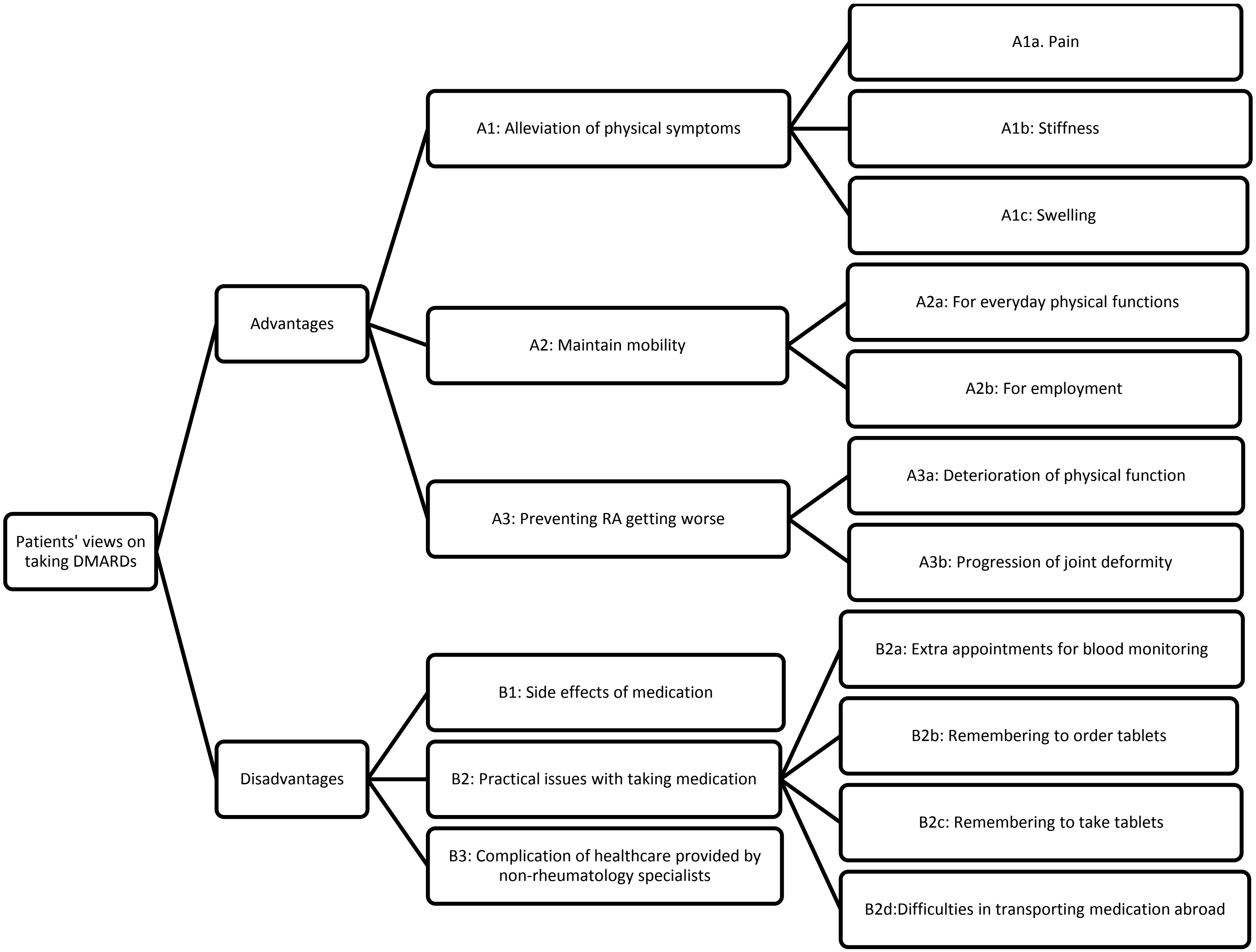

Supplement: Supplementary file 2 — Figure S1. Hierarchial tree of analytic themes relating to advantages and disavantages of DMARD therapy. (PDF 129 kb) [file 41927_2019_70_MOESM2_ESM.pdf]
