# Supplementary material for: “Living a normal life”: a qualitative study of patients’ views of medication withdrawal in rheumatoid arthritis
Source: BMC Rheumatol. 2019 Jun 13;3:2. doi: 10.1186/s41927-019-0070-y (PMC6567658; doi:10.1186/s41927-019-0070-y)

Supplementary Figure S2 – Hierarchical tree of analytic themes relating to withdrawal of DMARD therapy. Modifiers (M) that strengthen themes are shown in red, whereas modifiers that weaken themes are shown in blue.

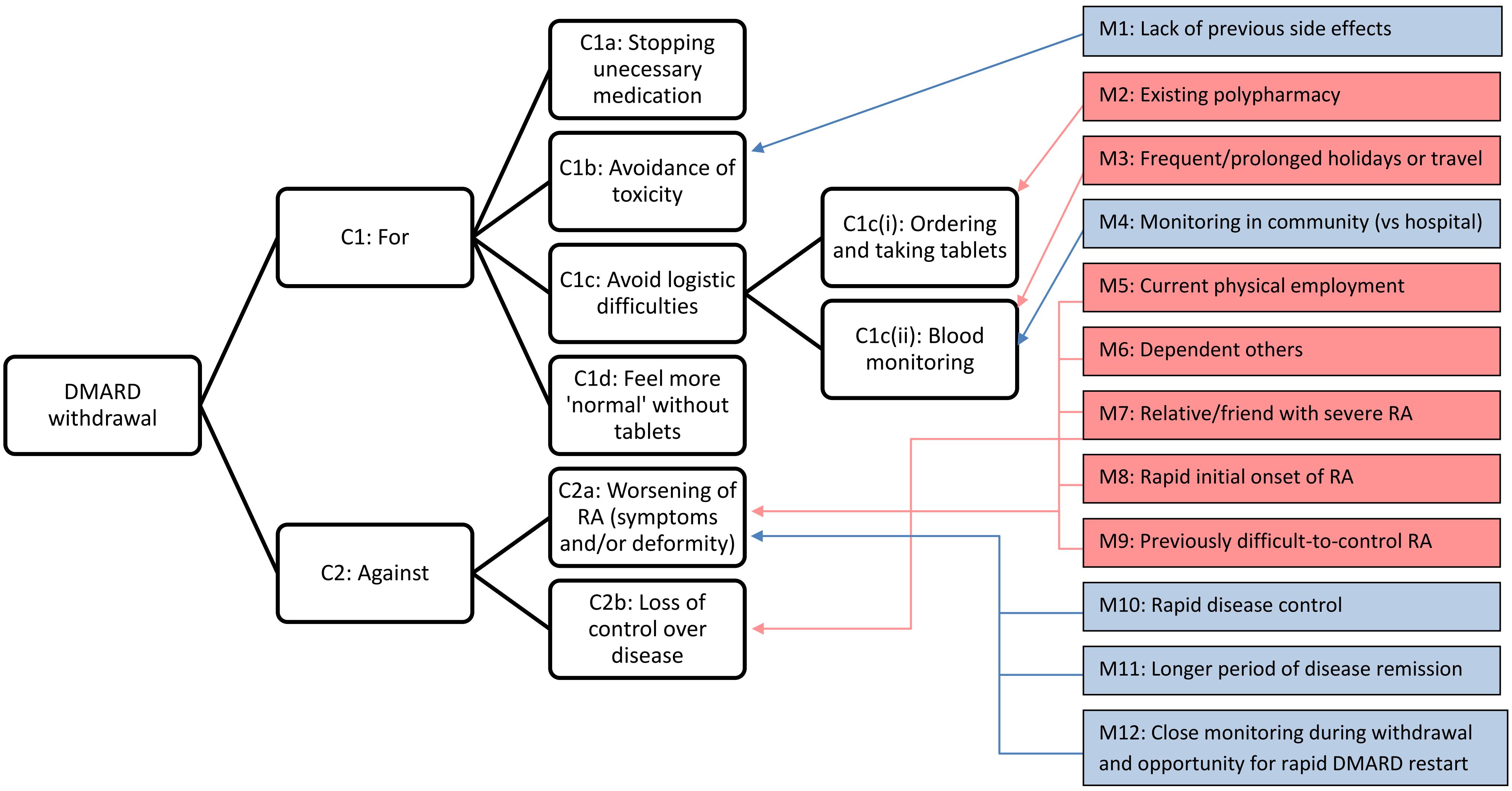

Supplement: Supplementary file 3 — Figure S2. Hierarchical tree of analytic themes relating to withdrawal of DMARD therapy. (PDF 129 kb) [file 41927_2019_70_MOESM3_ESM.pdf]
